# Determination of enriched histone modifications in non-genic portions of the human genome

**DOI:** 10.1186/1471-2164-10-143

**Published:** 2009-03-31

**Authors:** Jeffrey A Rosenfeld, Zhibin Wang, Dustin E Schones, Keji Zhao, Rob DeSalle, Michael Q Zhang

**Affiliations:** 1 Cold Spring Harbor Laboratory, Cold Spring Harbor, NY 11724 USA; 2 Department of Biology, New York University, New York, NY USA; 3 American Museum of Natural History, New York, NY USA; 4 Laboratory of Molecular Immunology, National Heart, Lung and Blood Institute, NIH, Bethesda, MD USA

## Abstract

**Background:**

Chromatin immunoprecipitation followed by high-throughput sequencing (ChIP-seq) has recently been used to identify the modification patterns for the methylation and acetylation of many different histone tails in genes and enhancers.

**Results:**

We have extended the analysis of histone modifications to gene deserts, pericentromeres and subtelomeres. Using data from human CD4^+ ^T cells, we have found that each of these non-genic regions has a particular profile of histone modifications that distinguish it from the other non-coding regions. Different methylation states of H4K20, H3K9 and H3K27 were found to be enriched in each region relative to the other regions. These findings indicate that non-genic regions of the genome are variable with respect to histone modification patterns, rather than being monolithic. We furthermore used consensus sequences for unassembled centromeres and telomeres to identify the significant histone modifications in these regions. Finally, we compared the modification patterns in non-genic regions to those at silent genes and genes with higher levels of expression. For all tested methylations with the exception of H3K27me3, the enrichment level of each modification state for silent genes is between that of non-genic regions and expressed genes. For H3K27me3, the highest levels are found in silent genes.

**Conclusion:**

In addition to the histone modification pattern difference between euchromatin and heterochromatin regions, as is illustrated by the enrichment of H3K9me2/3 in non-genic regions while H3K9me1 is enriched at active genes; the chromatin modifications within non-genic (heterochromatin-like) regions (e.g. subtelomeres, pericentromeres and gene deserts) are also quite different.

## Background

The chromatin state and transcription level of a chromosomal region has been found to be related to modifications of histones [1,2] as well as DNA [3,4]. The fundamental structural unit of chromatin is the nucleosome, which is formed by wrapping DNA around a histone octamer consisting of two copies each of four core histone proteins (H2A, H2B, H3, and H4). The tails of these histones can be modified in a variety of ways that relate to chromatin condensation and gene expression[2]. Different histone residues can be methylated, acetylated, phosphorylated or ubiquitinated to either directly change the chromatin structure or allow for the binding of specific transcription factors [5].

The acetylation and methylation states of various lysine residues have been extensively investigated. Due to the reactive nature of the amine group on the side chain of lysine, it can be acetylated or mono-, di- or tri- methylated. Enrichment for acetylated histones has been found to correlate with euchromatic and gene-coding regions [6,7]. In contrast, certain methylations are associated with activation and others with repression. The most extensively characterized activating methylation is H3K4[8-10], while the H3K9 [11-13] and H3K27 [14-16] methylations are thought to lead to repression and inactivation. Recently, though, mono-methylations of H3K27 and H3K9 have been found to be enriched in active genes [17].

Using antibodies with specificity for a particular methylation state, cells have been profiled separately for mono-, di-, and tri- methylation of the same residue. These comparisons were originally performed using mass-spectroscopy and comparisons of staining patterns[18-20]. Since these studies relied on comparisons of staining patterns, they were only able to produce low-resolution results that could not be linked to sequence positions. More recently, the ChIP-chip [21] and ChIP-seq [17] techniques have been utilized for high-resolution sequence level profiling of histone modifications in mammalian genomes[22,17]. These techniques utilize chromatin immunoprecipitation coupled with either tiling microarray hybridization or high-throughput sequencing to determine the genomic locations of a particular modification. It is now possible to obtain resolution on the level of individual nucleosomes. Because histone modifications act on individual nucleosome and there may be modification differences between adjacent nucleosomes, this level of resolution is highly informative.

The most comprehensive profiling of histone modifications has been performed in CD4^+ ^T cells[17,23]. The genome-wide locations at a single nucleosome level resolution of 38 different histone methylations and acetylations were profiled, including different levels of methylation of the same residue. In these studies, the modification profiles surrounding the promoters of genes with different levels of expression were examined. Basic comparisons between the different modifications were performed in the region surrounding annotated genes, but here we are reporting the results of an extension of our initial studies [17,23] to non-genic regions of the human genome.

In particular, we wanted to investigate histone modifications in three important largely non-coding regions of the genome: pericentromeres, subtelomeres, and gene deserts. These regions are mostly non-coding, but little work has been performed to characterize and differentiate them with regard to histone modifications. Using the identified genome-wide locations for histone methylations and acetylations in CD4^+ ^T cells, we have identified the modifications that are strongly enriched in pericentromeres, subtelomeres, and intergenic regions (we have excluded all known genes in those regions). We have also used consensus repeat sequences to analyze histone modifications present in the unassembled portions of telomeres and centromeres.

## Results

### Characterization of histone modifications enriched in pericentromeres, subtelomeres or gene deserts

**Figure 1:**
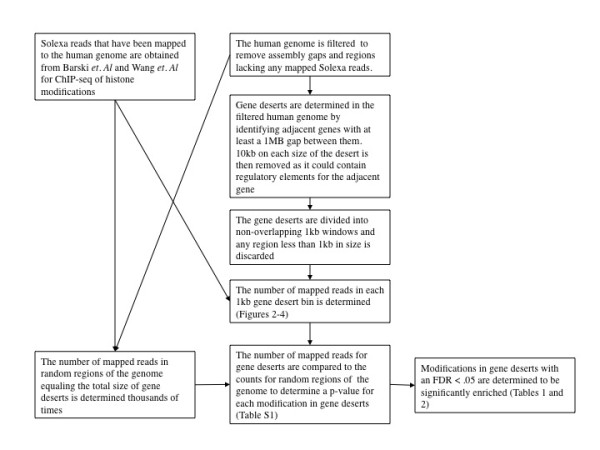
**A diagram outlining the analysis that was performed on the data**. The diagram uses the example of gene deserts to describe the analysis, but the same techniques were used for pericentromeres, subtelomeres and silent, medium expression and high expression genes.

To characterize the histone modifications that are enriched in different regions of the genome, we first obtained the locations of each type of region. Our determinations were made based upon gene annotations (see Methods and Figure 1 for details). Pericentromeres were defined for each chromosome as the region between the two genes most proximal to the centromeric gap in the genome assembly. From this region, the 10 kb nearest to each of the genes was removed since it could contain regulatory elements for the genes. Subtelomeres designations from [24] were used as a starting point. If a gene was found in a subtelomere, the gene was removed along with the 10 kb surrounding it on each side. Gene deserts were defined as regions of greater than 1 Mb between two adjacent genes, with the 10 kb proximal to each gene removed. From all three types of regions, any known Pol II binding sites[17] or DNase I hypersensitive sites [25] with 100 bp flanking regions were removed in order to exclude regions that may be related to the adjacent genes.

In each of these genomic regions, we determined the statistically significant histone modifications. First, the average number of tags for each modification in each type of region was determined. Random samples of the genome having the same size as each type of region were then taken and the number of tags for each modification was determined for the samples. By comparing the actual count to the counts in the random samples, an empirical p-value for significance could be determined (see methods for details). The results of this analysis are shown in Table 1. All of the modifications shown are significant with a q-value < 0.05, with the full data listed in Additional File 1.

**Table 1:** Significant Modifications in Genomic Regions

Pericentromeres	H3K9me2, H3K9me3, H3R2me2, H4K20me3, H4R3me2
Gene Deserts	H3K9me2, H3K9me3

Subtelomeres	H2AK5ac, H3K14ac, H3K27me2, H3K27me3, H3R2me1

Statistically significant modifications in pericentromeres, telomeres and gene deserts. Modifications listed are significant at a q-value of 0.05, with the full data listed in Additional File 1.

The results for pericentromeres are intriguing. The enrichment of H3K9me3 is expected, as it is a known mark for constitutive heterochromatin. H4K20me3 is a known mark of repressed chromatin and therefore it would be expected to be enriched in non-genic regions of the genome. Surprisingly, it is only found strongly in pericentromeres and in no other type of non-genic region. The appearances of H4R3me2 and H3R2me2 as significant modifications in pericentromeres are likewise interesting. Methylation of H4R3 has been generally correlated with expression and active chromatin [26,27]. But, as with other histone modifications, no distinction has been made between the two methylation states of H4R3, H4R3me1 and H4R3me2. Therefore, H4R3me2 could be a signal of pericentromeres while H4R3me1 could be a signal of active genes. This is supported by the fact that H4R2me2 levels were not found to have a preferential enrichment at either active or silent promoters [17]. H3R2me2 has been found to impede the binding of effector proteins to H3K4me3 and thus to control gene expression [28]. It is possible that the significant levels of H3R2me2 in pericentromeres are important in preventing the expression of genes in those loci. These four modifications are all strongly correlated with each other within pericentromeric regions indicating that they are likely to be found on the same nucleosomes.

For gene deserts, the di- and tri methylations of H3K9me3 were found to be significantly enriched, as would be expected [29]. Distinctly lacking, were H3K27me3 and H3K9me1. This absence could be due to the presence of H3K27me3 and H3K9me1 in silent and active genes (see below). For subtelomeres, the repressive marks H3K27me2/3 were found to be enriched along with two acetylations that are marks of activation, H2AK5ac and H3K14ac.

### H4K20 and H3K9 methylation levels are varied in pericentromeres, subtelomeres and gene deserts

**Figure 2:**
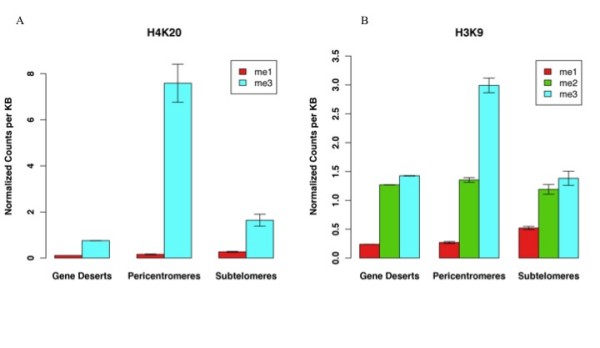
**A comparison of the enrichment of different levels of methylation at the same residue in gene deserts, pericentromeres and subtelomeres**. The mean has been plotted and the error bars show plus and minus one standard error of the mean. All of the data has been normalized to the number of counts of each modification in the genome, so the genomic average for each mark is 1.

To further characterize the histone modifications in different non-genic regions, we compared the multiple methylation states of individual residues in each region. The number of tags identified for each methylation state of H4K20 and H3K9 in pericentromeres, subtelomeres and gene deserts were counted and the results are shown in Figure 2. For H4K20me, the differences between the types of chromatin are significant. In each region, levels of H4K20me1 are minimal and invariant. One possible reason for this is that H4K20me1 is a marker of active genes [17,30] and it is depleted in constitutive heterochromatin. The reverse is found for H4K20me3 which has been found to associate with constitutive heterochromatin [31]. In pericentromeres, the level of H4K20me3 is over 40 times the level of H4K20me1. This difference decreases in gene deserts and it is lowest in subtelomeres where the level of H4K20me3 is only double the level of H4K20me1. This indicates that H4K20me3 may be important for pericentromeres.

We next compared the three levels of methylation of H3K9 (Figure 2B). The levels of H3K9me2/3 have been found to be enriched in non-genic regions, while H3K9me1 levels are enriched at active genes [17]. We found that for each type of non-genic region the order of enrichment levels is the same with H3K9me3> H3K9me2>H3K9me1. While the order is the same, the ratio between the methylation levels varies. In pericentromeres, the levels of H3K9me3 are almost double those of H3K9me2. This difference is greatly decreased in subtelomeres where the levels are similar and within one standard error of the mean (see error bars). In pericentromeres, the combined levels of H3K9me2 and H3K9me3 are 11 times those of H3K9me1. This is reduced to 4 times the level of H3K9me1 in subtelomeres.

### Analysis of histone modifications at centromeric and telomeric repeats

Since large regions of the centromeres and telomeres of the human genome are not included in the genome assembly, they could not be accurately probed by our techniques. To probe these regions, we have used a technique similar to that previously used for studying histone modifications at repetitive regions in the mouse genome [32]. As a proxy for the actual genome sequences at repetitive locations, consensus repeat sequences are used. We have taken all of the reads for each methylation and determined the percentage of them that aligned either to telomere or centromere consensus repeat sequences. Since the percentages of repeats were calculated and compared, no normalization for differences in antibody specificity or number of sequenced tags were required. The percentage is a self-normalizing calculation. (see Methods for details)

For centromeres, the most represented modifications are H4K20me3 and H3K9me3 and the least significant modifications are H3K4me1 and H4K20me1. Both H4K20me3 and H3K9me3 are known to be associated with constitutive heterochromatin[29,33] and were also found to be significantly enriched in pericentromeres. This indicates that centromeres have heterochromatic characteristics and may require these histone modifications to prevent expression. For telomeres, the most significant modifications are H2BK5me1 and H3K4me3 and the least significant are H3K36me3 and H3K9me3. The finding of these two activating modifications to be the strongest in telomeres is interesting and is likely to be related to the recent finding of RNA polymerase II transcription at mouse[34] and human[35] telomeres. The lack of H3K9me3 also indicates that telomeres are not consistent with other non-coding regions of the human genome. In contrast to the middle of a chromosome where pericentromeres and centromeres have similar enriched histone modifications, the ends of chromosomes at subtelomeres and telomeres have different modifications. We also found that a much larger percentage of the tags matched the centromeric repeats than the telomeric repeats. The percentage of tags matching the centromeric repeats range from 0.02% to 0.8%, while the range for telomeric repeats is from 10e^-4^% to 10e^-3 ^%. This can be explained by the much larger percentage of the genome contained in centromeres than in telomeres [36,37]. Because the percentage of reads was compared rather than the raw numbers of reads, this result should not be affected by differences in sequencing depth.

### Determination of modifications with statistically significant enrichment in different regions of the genome

We next decided to compare our findings for non-genic regions to the genic portions of the genome. Using comparisons to random samples of the genome (see above and methods), we determined the histone modifications statistically significant in silent genes, medium expression genes, high expression genes, and a combined set of all genes without regard to expression. The complete list of significant modifications is summarized in Table 2. All modifications listed in this table are significant at a q-value of 0.05 and a complete list of all modifications with their mean counts and empirical p-values and q-values are in Additional File 1. When all of the genic regions without regard to expression were tested, all of the acetylations and some of the methylations were found to be significant. Interestingly, when the genes were divided into groups by expression level, different modifications were found to be significant for each level of expression. The least well-characterized groups are silent genes and non-genic regions which each only have a few modifications significantly enriched. In contrast, there are many modifications that are preferentially associated with active genes.

**Table 2:** Significant Modifications in Genes with different levels of expression and Non-genic Regions

High Expression Genes	H2AK5ac, H2AK9ac, H2AZ, H2BK120ac, H2BK12ac, H2BK20ac, H2BK5ac, H2BK5me1, H3K14ac, H3K18ac, H3K23ac, H3K27ac, H3K27me1, H3K36ac, H3K36me3, H3K4ac, H3K4me1, H3K4me2, H3K4me3, H3K79me1, H3K79me2 H3K79me3 H3K9ac, H3K9me1, H3R2me1, H3R2me2 H4K12ac, H4K16ac, H4K20me1, H4K5ac, H4K8ac, H4K91ac
Medium Expression	H2AK5ac, H2AK9ac, H2AZ, H2BK120ac, H2BK12ac, H2BK20ac, H2BK5ac, H2BK5me1, H3K14ac, H3K18ac, H3K23ac, H3K27ac, H3K27me1, H3K27me2, H3K27me3, H3K36ac, H3K36me3, H3K4ac, H3K4me1, H3K4me2, H3K4me3, H3K79me1, H3K79me2, H3K79me3, H3K9ac, H3K9me1, H3R2me1, H3R2me2, H4K12ac, H4K16ac, H4K20me1, H4K20me3, H4K5ac, H4K8ac, H4K91ac, H4R3me2

Silent Genes	H2AZ, H3K27me2, H3K27me3, H3K9me2

Non-genic Regions	H3K9me2, H3K9me3

Statistically significant modifications in different regions of the genome. Modifications listed are significant at a a-value of 0.05, with the full data listed in Additional File 1.

### H3K9me2/3 and H3K9me1 have variant levels of enrichment in non-genic regions and in genes

Since the significance analysis found H3K9me2 and H3K9me3 to be enriched in non-genic regions and H3K9me1 enriched at active genes, we wanted to further investigate the relationship between H3K9 methylation state and expression level. In general, H3K9 methylation has been considered to be an indicator of inactive and silent chromatin[13,11,12], but increased levels of H3K9me1 are found consistently in the gene body of active genes [17]. Interestingly, certain active genes have also been found to have H3K9me3[38-40].

**Figure 3:**
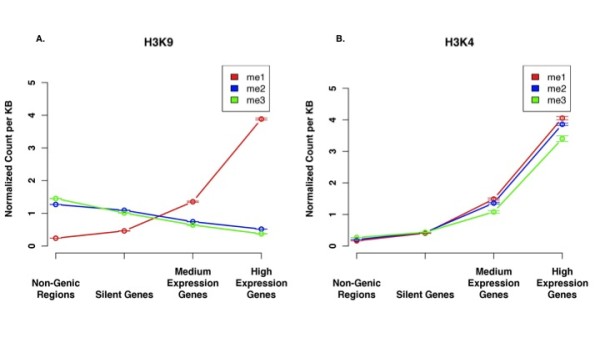
**A. A comparison of the profiles of the different levels of methylation of H3K9 from non-genic regions to high expression genes**. The points represent the mean value with the error bars showing the standard error. **B**. A comparison of the profiles of the different levels of methylation of H3K4 from non-genic regions to high expression genes. All of the data has been normalized to the number of counts of each modification in the genome, so the genomic average for each mark is 1.

To investigate the roles of mono-, di- and tri-methylated H3K9, we compared the levels of enrichment of each modification in overall non-genic regions in addition to genes with different levels of activity (Figure 3A). It was found that the overall profiles for H3K9me2 and H3K9me3 are highly similar (Pearson correlation of 0.99). The enrichment for these two modifications was highest in non-genic silent regions and decreased as the activity of the regions increased. In contrast, a completely opposite profile was found for H3K9me1. As the activity level for genomic regions increased, the level of H3K9me1 increased greatly. These findings indicate that H3K9me is not a monolithic modification and H3K9me1 is distinct from H3K9me2/3.

### The three levels of H3K4 methylation are all activating marks, but H3K4me3 is the least enriched in active genes relative to inactive genes

H3K4me has long been considered an activating mark for chromatin, but differences between H3K4me2/3 and H3K4me1 have been found[8,17,41]. To compare the enrichments of H3K4me1, H4K4me2, and H3K4me3 in broader regions, we compared the counts of each modification in regions of the genome (Figure 3B). This figure suggests that the enrichment patterns for H3K4me1, me2, and me3 are almost congruent (Pearson correlation of 0.99) with each other indicating that all three levels of methylation of H3K4 are equally important determinants of expression level as activating marks. The only regions of the genome having a significant difference between the modifications of H3K4 are highly expressed genes. In these regions H3K4me1 and H3K4me2 are enriched at higher levels than H3K4me3

### The methylation states of H3K27 each have a distinct profile

**Figure 4:**
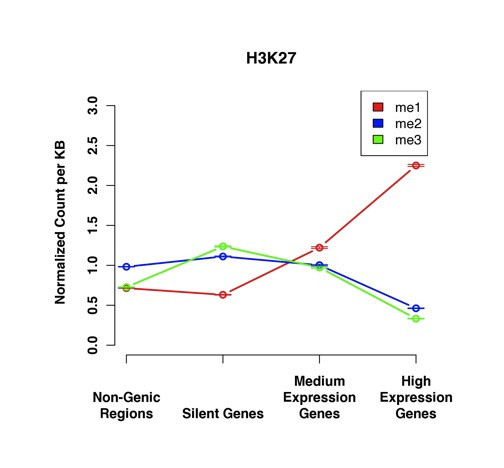
**Profiles of different levels of methylation for H3K27**. The points represent the mean value with the error bars showing the standard error. Each level of methylation of this residue has a distinct profile with the tri-methylation most highly enriched in silent genes. Even though the levels of the three types of H3K27me are indistinguishable in silent genes, the levels of H3K27me3 are clearly higher there than anywhere else. All of the data has been normalized to the number of counts of each modification in the genome, so the genomic average for each mark is 1.

Different methylation states of H3K27 are found to be significant in distinct parts of the genome (Table 2). Because of this variation, we wanted to determine how the different modification states are related to genes with different levels of expression. We determined the number of counts for each methylation state of H3K27me3 in each type of region in the genome. The resulting plots are shown in Figure 4.

It was found that the levels of H3K27me3 are most enriched at silent genes. The levels of this modification is similarly minimal in both non-genic regions and active genes. Rather than having an enrichment level that is either correlated or anti-correlated with expression, it has a parabolic profile with the peak in silent genes. This finding is especially interesting when considered in concert with the profiles of the other levels of methylation of H3K27 and their presumed functions. Each level of methylation of these two residues has a completely distinct profile. This diversity is much greater than what was found for any other histone residues including H3K4, H4K9 and H4K20.

## Discussion

We have analyzed histone modification patterns in pericentromeres, subtelomeres, and gene deserts. It was found that each region has a distinct histone modification profile even though they are all traditionally considered to be constitutive heterochromatin or heterochromatin-like [42]. This is not unexpected since each of them has a distinct function. Pericentromeres are involved in the cohesion of sister chromatids during cell division [43], subtelomeres maintain chromosome stability [24,44] and gene deserts do not have a well defined function but they may contain long-range regulators of gene function [45,46] or structural regions such as the nuclear matrix attachment sites [47,48]. Each of these functions is performed by a specific set of DNA binding proteins and thus should be associated with distinct histone modifications. One possibility for the discrepancy between H3K9me3 levels in gene deserts and pericentromeres as compared to telomeres relates to their locations in the genome. Pericentromeres and gene deserts are in the middle of a chromosome between genes (even though the genes may be at a great distance) and therefore they need H3K9me3 which will interact with HP1 [49] to prevent transcription from occurring. Subtelomeres do not require such an enrichment of H3K9me3.

A further conclusion from the results in Table 1, is that the modifications found in gene deserts are a subset of the modifications that are found at pericentromeres. Both contain repressive H3K9me2/3, but pericentromeres also have H3R2me2, H4K20me3 and H4R3me2. This difference may be due to the replication associated functions of pericentromeres [50]. This is in addition to the requirement of a constitutive heterochromatin sequence at centromeres [43].

A recent study [32] looked at constitutive heterochromatic regions of the genome by aligning ChIP-seq reads to consensus sequences for repetitive elements and found that H3K9me3 and H4K20me3 were strongly enriched. We have reached a similar conclusion, but were able to separate the centromeric and telomeric regions and distinguish the modifications that are enriched in each of these regions. We found that the enrichment of H3K20me3 in non-coding regions is largely due to its high level in pericentromeres and that it is found at lower levels in subtelomeres and gene deserts.

The comparisons between non-coding regions and genes with different levels of expression have allowed us to determine the histone modifications enriched in each region. We have found that silent genes have a distinct histone modification profile that is not merely an intermediate between genes with high levels of expression and non-genic regions. Non-coding regions are characterized by high levels of H3K9me2/3 whereas silent genes are characterized by H3K27me3. This is contrary to previous studies that have grouped H3K9me2/3 and H3K27me3 as repressive modifications [29,51] without identifying significant differences between them. Our higher resolution and sharper distinction between the genomic regions allowed for this differentiation.

## Conclusion

We have found that regions of the genome that do not contain actively expressed genes do not have a monolithic histone modification profile. Silent genes as well as different sub-categories of non-coding regions including gene deserts, subtelomeres and pericentromeres each have different significantly enriched histone modifications. We have also determined the histone modifications at highest levels in non-assembled telomeres and centromeres. Additionally, we have found that for each histone residue studied different methylation states are enriched in different parts of the genome. It is therefore an oversimplification to speak about the function of methylation of a histone residue without specifying the methylation state under discussion.

## Methods

### Chromatin modification data

The chromatin modification data was obtained from [23,17]. The histone modifications analyzed were: H2AK5ac, H2AK9ac, H2AZ, H2BK120ac, H2BK12ac, H2BK20ac, H2BK5ac, H2BK5me1, H3K14ac, H3K18ac, H3K23ac, H3K27ac, H3K27me1, H3K27me2, H3K27me3, H3K36ac, H3K36me3, H3K4ac, H3K4me1, H3K4me2, H3K4me3, H3K79me1, H3K79me2, H3K79me3, H3K9ac, H3K9me1, H3K9me2, H3K9me3, H3R2me1, H3R2me2, H4K12ac, H4K16ac, H4K20me1, H4K20me3, H4K5ac, H4K8ac, H4K91ac, H4R3me2.

### Determination of genomic regions and types of chromatin

The genic regions of the genome were the UCSC Known Genes (April 2007 release). To remove redundancy, splice variants of genes were combined and each exon was only included once in the set. Additionally, only genes that had accurate unambiguous mappings to Affymetrix U133 Plus 2.0 arrays were used. This procedure resulted in a list of 12,544 genic regions. For all of the analyses, the gene region refers to all of the sequence from the start to end of transcription.

The genes were divided into three groups based upon their expression level in CD4^+ ^cells[52]. The high expression genes are the 1000 genes with the highest level of expression, the medium expression genes are the middle 1000 genes when the genes are sorted by expression and the silent genes are the 1000 genes with the lowest level of expression.

The gene desert regions were determined by first identifying adjacent genes in the genome with a 1 Mb break between adjacent genes. From these regions, the outer 10 kb on each end that could contain regulatory elements for the neighboring genes were excluded. To further purify the sequence, any known DNase hypersensitive sites[25] and RNA Pol II binding sites[17] were removed along with 100 bp flanking regions on each side of these sites

The subtelomeric regions were taken from[53]. The original data from that manuscript  contained genomic coordinates for the July 2003 (hg16) build of the human genome. These coordinates were mapped onto the Mar. 2006 (hg18) assembly using the UCSC LiftOver tool. Of the 21 Mb of sequence that were identified in hg16, 13 Mb could be accurately mapped to hg18.

The pericentromeric regions were determined based upon the centromere locations identified in the UCSC Genome Browser. The sequence between the two most proximal genes on either side of the pericentromere was obtained. From this sequence, any known DNase hypersensitive sites [25] and RNA Pol II binding sites [17] were removed along with 100 bp flanking regions on each side of these sites.

In order to refine the regions that were being used and to eliminate the number of false negative that were found, we utilized the identified nucleosome locations within the human genome [52] as a control. For a histone to be modified, there needs to be a nucleosome present, thus any region in the genome that is lacking nucleosomes by definition, must lack all histone modifications. In addition, there are regions throughout the human genome that cannot accurately be mapped accurately using the short reads [54,55]. We therefore only included those regions of the genome in which nucleosomes were found. We mapped all of the nucleosome reads from [52] to the human genome and then extended the read length to 150 bp to include the entire length of the nucleosome. This resulted in an effective genome size of 2,171,857,609 bp. The percentages of each type of region that was covered by nucleosomes and therefore analyzed are in Additional File 2.

### Determination of counts in particular regions

The determination of the number of counts for a particular modification in a genome region will be outlined using the example of H3K4me3 and high expression genes. This same method was used for each modification and each type of genomic region. All of the high expression genes were extracted and divided into non-overlapping 1 kb windows. For each window, the number of H3K4me3 tags whose middle was within the window was counted. This count was then normalized by dividing it by the average number of counts for H3K4me3 in 1 kb windows in the whole genome covered by nucleosomes. Through this normalization step, the counts of different modifications were made comparable. The raw counts are affected by differences in the affinities between antibodies for different modifications and therefore have different distributions. A normalized count was determined for each window and the mean and standard error of the mean were calculated. This mean appears in the graphs as the normalized count per kb for H3K4me3 in high expression genes. The standard error of the mean is shown in the error bars.

### Statistical Significance of Modifications

The average counts of each modification in all regions of a particular type were compared with random samples of the nucleosome filled portions of the genome (see above). The rank of the actual count in the distribution of the randomized counts gave an empirical p-value. Using the example of H3K4me3 in high expression genes this will be illustrated. The total count of H3K4me3 marks in the entire gene body from the TSS to the end of transcription for all high expressed genes was determined. Then, ten thousand random samples of the nucleosome-filled genome each having the same total number of bases as that of all of the high expressed genes were made. For each random sample, the total number of counts of H3K4me3 was determined. These sample counts were used to create a null distribution for the number of H3K4me3 tags that would be found in a random portion of the genome covered by nucleosomes. The actual count was then compared to the null distribution to calculate an empirical p-value. In essence, this randomization determined how strongly H3K4me3 was enriched in high expressed genes relative to genes with lower levels of expression and non-coding regions. The p-values that were produced from this randomization were corrected for multiple testing using a false-discovery rate (FDR) technique [56,57]. All modifications that are marked as significant in a particular region of the genome are significant at a q-value of 0.05.

### Centromeric and Telomeric Repeats

For centromeric and telomeric repeats, the consensus sequences in RepBase [58] were used. All of the tags for each modification were collected and compared to the specified repeat sequence using RMAP [55]. Since RMAP is designed as a program for aligning short sequencing reads to a genome, it needed to be used in a modified fashion. The "genome" that the reads were compared to was the consensus sequence for either centromere or telomere repeats. Thus, running RMAP with the entire list of H3K9me3 reads and the telomere repeat sequence, would give the number of H3K9me3 reads that mapped to the telomere repeat. This count was then divided by the total number of H3K9me3 reads to give the percentage of reads mapping to telomeres. A percentage was used in order to correct for differences between the read libraries for the different histone modifications that could be affected by many different factors including antibody specificity and the number of sequencing runs performed. If there were twice as many reads for modification A as for modification B, the percentages would still be directly comparable because modification A would have twice as many total reads along with twice as many telomere reads which would produce the same percentage or mapped reads.

## Authors' contributions

JAR, RD, KZ and MQZ conceived of the study and participated in its design. ZW and DES assisted in the preparation and analysis. JAR an MQZ wrote the manuscript. All of the authors have read and approved the manuscript.

## Supplementary Material

Additional file 1 Significance of all studied histone modifications throughout the genome. The full dataset from which Tables 1 and 2 were derived. For each modification in each type of genomic region the mean counts per kb, standard error of counts, the empirical p-value and the FDR rate (q-value) are listed. Click here for file

Additional file 2 Percentage of genomic regions utilized in this study. A breakdown of genomic regions. The blue regions are gaps in the genomic alignment; green regions are regions lacking reads for any of the antibodies used. Green regions are those regions with a size less than 1 kb. All of these regions were discarded and not used. The remaining yellow regions were used for the an Click here for file
