# Supplementary figures and images for: Determination of enriched histone modifications in non-genic portions of the human genome
Source: BMC Genomics. 2009 Mar 31;10:143. doi: 10.1186/1471-2164-10-143 (PMC2667539; doi:10.1186/1471-2164-10-143)

Pericentromeres

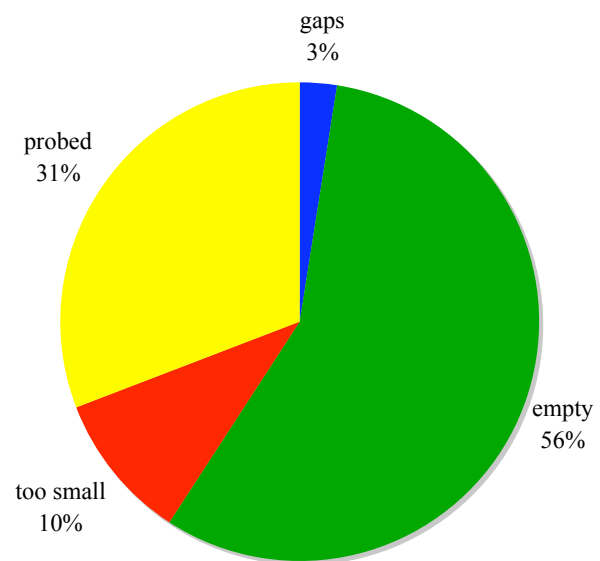

Subtelomeres

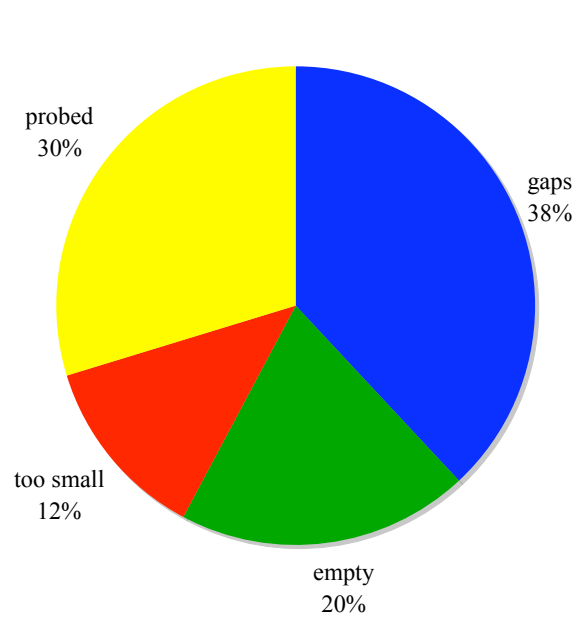

Gene Deserts

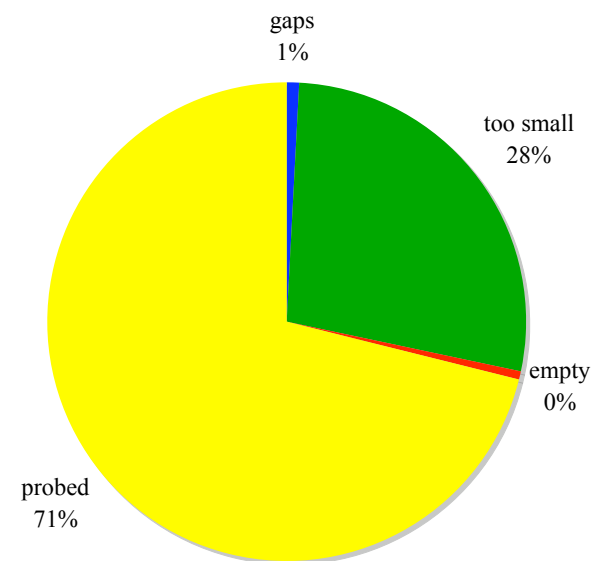

Supplement: Additional file 2 — Percentage of genomic regions utilized in this study. A breakdown of genomic regions. The blue regions are gaps in the genomic alignment; green regions are regions lacking reads for any of the antibodies used. Green regions are those regions with a size less than 1 kb. All of these regions were discarded and not used. The remaining yellow regions were used for the an [file 1471-2164-10-143-S2.pdf]
